# Influenza Virus-Associated Acute Necrotizing Encephalopathy in Two Young Children: Case Report

**DOI:** 10.3390/reports7040118

**Published:** 2024-12-22

**Authors:** Prisca Largo, Olivia C. Arnone, Francesco Sacco, Gaetano Cantalupo, Paolo Biban

**Affiliations:** 1 Pediatric Intensive Care Unit, University Hospital of Verona, Piazzale Stefani 1, 37126 Verona, Italy; prisca.largo@aovr.veneto.it (P.L.); francesco.sacco@aovr.veneto.it (F.S.); 2 School of Pediatrics, University of Verona, 37126 Verona, Italy; oliviarnone@gmail.com; 3 Child Neuropsychiatry Unit, Department of Children and Maternal Health, AOUI Verona, Full Member of European Reference Network EpiCARE, 37126 Verona, Italy; gaetano.cantalupo@univr.it

**Keywords:** influenza virus infection, acute necrotizing encephalopathy, MRI cerebral scan, high-dose corticosteroids

## Abstract

**Background and Clinical Significance**: Acute necrotizing encephalopathy (ANE) represents a severe complication, mainly described in children, of influenza virus infection. We report the cases of two young girls with ANE associated with influenza virus infection who were diagnosed by MRI cerebral scan. **Case Presentation**: A 7-year-old girl with a history of a previous episode of ANE presented with a worsening drowsy state and seizures. In the second case, an otherwise healthy 5-year-old girl presented with fever, seizures, and marked neurological deterioration. In both cases, nasopharyngeal swab testing was positive for influenza virus A, while cerebral MRI indicated ANE. Despite aggressive treatment with high-dose corticosteroids and a five-day course ofimmunoglobulins, the ultimate prognosis was poor in both patients. ANE is a serious complication of viral infections in children, with a high mortality rate and a broad spectrum of neurological sequelae. To date, the pathophysiology and management of influenza virus-induced ANE remain uncertain. Although ANE is usually sporadic, familial and recurrent cases have been reported, and anRAN-binding protein (RANBP2) mutation has occasionally been associated with its occurrence.Conclusions: Rapid recognition of neurological symptoms and suspicion of a viral trigger, especially in influenza-like illnesses, are both essential for the timely administration of effective therapy. Further research is needed to clarify the pathophysiology of ANE and establish the best therapeutic strategies to fight such a deadly disease.

## 1. Introduction and Clinical Significance

Influenza A and B viruses cause yearly seasonal epidemics that are associated with high rates of hospitalization and significant morbidity and mortality [1]. These infections are particularly common in children, who in turn spread the viruses within their social environments, leading to high rates of school absences and a socioeconomic burden [2].

The main symptoms of children infected with influenza are fever, cough, rhinorrhea, coryza, pharyngitis, and headache. Less common symptoms include myalgia and muscle fatigue. Compared to adults, children present higher rates of gastrointestinal symptoms, including diarrhea, vomiting, and abdominal pain [2].

Although most individuals recover from the primary infection in a few days, some children may suffer from complications, including pneumonia, secondary bacterial infections such as sinusitis or acute otitis media, worsening of pre-existing respiratory conditions, and seizures [3].

Rarely, influenza-associated forms of encephalopathy/encephalitis can also be observed. Among these, acute necrotizing encephalopathy (ANE) represents the most severe form, mostly described in the pediatric population [4]. ANE is characterized by a high mortality rate (30–40%) [5] and variable neurological sequelae (40%), depending on the affected brain areas [5,6]. Less than 10% of children who survive the disease show a full recovery [7].

The main symptoms of ANE include rapidly fluctuating levels of consciousness, focal neurological deficits, and seizures. Brain magnetic resonance imaging (MRI) can detect inflammation of the central nervous system (CNS). Typical MRI findings include symmetrical bilateral lesions of the thalamus, periventricular white matter, brainstem, and cerebellum [8].

We report the clinical course of two young children with influenza virus-induced ANE who suffered severe sequelae despite treatment with high-dose corticosteroids and immunoglobulin therapy. Potential additional treatments for ANE will also be discussed.

Clinical Significance: The pathophysiology and specific management of influenza virus-associated ANE remain ill-defined. Current treatments consist of the administration of intravenous corticosteroids, immunoglobulins (IVIg), and antivirals, although their efficacy is uncertain, and morbidity and mortality rates remain high, especially in children with brainstem disease [9].

## 2. Case Presentation

### 2.1. Case 1

A 7-year-old Caucasian girl with no family history of encephalopathy presented to the pediatric emergency department (PED) with fever, fatigue, and worsening drowsiness.

Her past medical history revealed a previous status epilepticus at nine months of age, treated with carbamazepine, and an episode of necrotizing encephalopathy associated with a suspected bacterial infection at two years and eight months, treated with intravenous immunoglobulin therapy. A genetic test for mutations in RAN binding-protein 2 (RANBP2) was negative. After two months, the patient was hospitalized again for recurrent behavioral disorders associated with aggressive outbursts. Those episodes were interpreted as a possible acute-onset pediatric neuropsychiatric syndrome (PANS) and treated with benzylpenicillin, intravenous immunoglobulin, gabapentin, and risperidone. Afterwards, no further episodes of seizures were reported.

At PED admission, the Glasgow Coma Scale (GCS) was 8, the electroencephalogram (EEG) showed widespread slow brain activity, and the brain MRI was pathognomonic for acute encephalopathy. The MRI findings included specific hyperintense signal alterations in the long TR sequences and hypointensity in the T1 symmetrically in the posterior mesial portions of the thalami, both hippocampi, part of the splenium of the cerebral peduncles, the tegmentum, and the mesencephalic roof, as well as in the cerebellum. These structures were diffusely swollen, showing reduced diffusivity in several areas. The fluid attenuated inversion recovery (FLAIR) sequences showed blurred hyperintensity of the corticospinal tracts in the inner capsule–mesencephalon transition, in the pyramids, and in the white matter of the trigons. Due to her comatose state, the patient was admitted to our pediatric intensive care unit (PICU) and required invasive mechanical ventilation for a few days, followed by non-invasive ventilatory support after extubation. During hospitalization, repeated episodes of bradypnea, desaturation, and occasional bradycardia with spontaneous resolution were observed.

Her initial blood tests were normal, except for a slight increase in C-reactive protein (CRP = 9 mg/L) and transaminases (ALT 151 U/L).

A lumbar puncture showed increased protein and glucose levels, normal cell count, and negative cerebral spinal fluid (CSF) culture. Nasopharyngeal swab testing was positive for influenza type A-H3 virus. Rheumatological and metabolic investigations were negative.

Following the MRI findings, we administered high-dose corticosteroids (methylprednisolone at 30 mg/kg), followed by prednisone for tapering, with no clinical improvement. A follow-up MRI was repeated after five days, highlighting the reduction of the edematous components, but also the appearance of new lesions in the thalamus. Therefore, a five-day treatment with intravenous immunoglobulins was administered.

However, the patient remained in a neurovegetative state, while EEG monitoring showed a slight improvement during hospitalization. The patient was transferred to a specialized rehabilitation center in the following months, showing a gradual, albeit partial, recovery of some neurological functions.

### 2.2. Case 2

An otherwise healthy, 5-year-old Caucasian female presented at a District Hospital with fever, impaired sensorium (GCS 11), and seizures. There was no family history of encephalopathy. Intravenous midazolam was administered, with partial response. An urgent cerebral CT scan showed no specific alterations. Subsequently, the same symptoms occurred again, so phenytoin and acyclovir were administered to treat a suspected viral encephalopathy, with no clinical improvement. Hence, the patient was sedated, intubated, and transferred to our PICU.

**Figure 1:**
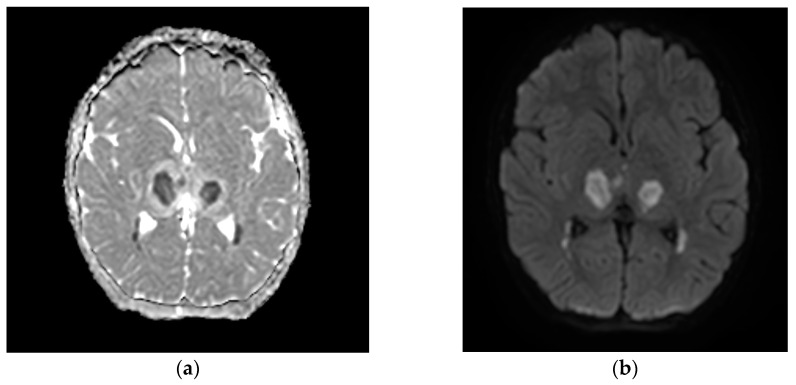
Typical MRI images of ANE in case 2: (**a**) low apparent diffusion coefficient (ADC) values; (**b**) restricted diffusion in the thalamus bilaterally.

A neuropsychiatric evaluation and video EEG excluded seizures. Despite sedation being progressively weaned and then stopped, severe neurological deterioration persisted, with a GCS of 6. A brain MRI showed features compatible with ANE, including multiple localized signal hyperintensity in the T2/FLAIR sequences, especially in the cortico-subcortical frontal areas, in both thalami, in the cerebral peduncles, and the hemispheric and vermian parts of the cerebellum (Figure 1). The white matter of the semioval centers and putamen were involved as well. Diffusivity restriction, with low apparent diffusivity coefficient (ADC) values, was observed for cortical localizations in the precentral gyrus bilaterally, in the right middle frontal gyrus and right entorhinal cortex, in the semioval centers, and the peri trigonal, putaminal, thalamic, pontine tegmentum, cerebellar hemispheric, and vermian cortical and subcortical sites.

A CSF sample was negative for infection, showing increased protein and glucose levels and a normal cell count. Nasopharyngeal aspirate tested positive for influenza type A-H1 virus and metapneumovirus. Transaminases (ALT) peaked at 2609 U/L and decreased progressively during hospitalization. Rheumatological and metabolic disorders were excluded.

In the suspected diagnosis of ANE, high-dose corticosteroids (methylprednisolone at 30 mg/kg) were administered, followed by prednisone for weaning. Subsequently, a five-day treatment with intravenous immunoglobulin was started.

Nonetheless, the patient’s neurological status remained unchanged, with hyporesponsiveness (GCS 7-8), photoreactive mydriatic pupils, and hypertonic upper and lower extremities.

After 19 days, the patient was transferred to the pediatric neuropsychiatric ward, where baclofen therapy was initiated to reduce the hypertonic status. The upper limbs’ somatosensory-evoked potentials (SEP or SSEP) showed regular conduction along the peripheral sensory pathway but no cortical response. The brainstem auditory evoked potential (BAEPs) and visual evoked potentials (VEPs) were normal. The patient was later discharged. Genetic tests for RANBP2 and its correlated genes were negative.

At a 6-month follow-up, the patient was in a wheelchair, with hypertonicity of the lower limbs but was able to follow simple orders and say a few words. She was orally fed with semi-solid foods.

## 3. Discussion

ANE is a well-recognized complication of influenza virus infection, causing an estimated 27% to 36% of childhood encephalopathy [10]. Clinical presentation of ANE is not specific and may include neurological deterioration and/or other organ dysfunctions. Sometimes, sudden sensory alteration can be the first symptom leading to medical evaluation, as was noted in both our patients [5]. The clinical course is characterized by progressive encephalopathy, with rapid onset of lethargy and often, seizures. Usually, a deep coma state requiring mechanical ventilation occurs early, from several hours to 1 day after the first neurologic symptoms. Laboratory findings are not specific, and CSF analysis reveals normal or mildly elevated protein levels and no isolation of influenza virus [5]. Characteristic MRI findings reveal multiple, symmetrical, and focal lesions of the thalami, with hyperintense signals, and less frequently, involvement of other brain areas, including cerebellum and brainstem lesions, as was observed in our two patients [8].

The pathogenetic mechanism of ANE has yet to be fully understood. Autoptic studies have shown necrosis, hemorrhages in the thalamus and pons, myelin pallor in the deep cerebral and cerebellar white matter or pathologic vascular endothelium, with vasogenic edema [6]. The non-detection of influenza virus in the central nervous system (CNS) has led to the hypothesis that the inflammation causing brain damage may originate outside the CNS [11]. This hypothesis is supported by other studies that reported higher cytokine levels in the cerebrospinal fluid compared to those measured outside the CSF. However, some authors have hypothesized that the influenza virus induces a marked inflammation that triggers a cytokine storm, disrupting the blood–brain barrier and leading to the necrotic brain lesions observed in ANE [11]. In particular, it has been found that interleukin 6 (IL-6) and tumor necrosis factor (TNF)-alpha are increased in severe cases of ANE, showing the significance of the inflammatory reaction in the pathogenesis of the brain damage typical in these types of encephalitis [12].

Although most cases of ANE seem sporadic, both familial and recurrent cases have been reported [13,14]. Interestingly, some forms of ANE have been associated with a mutation in the RANBP2, being referred to as ANE type 1. However, the role of this mutation in ANE remains unclear. Increasing evidence demonstrated that RANBP2 may interact with viruses to regulate viral infection [15]. Moreover, RANBP2 appears to regulate the innate immune response pathways and be implicated in different cellular processes, including energy maintenance in the neurons by regulating microtubule and mitochondrial function [13]. In ANE, cytokines may be produced within the CNS by microglial cells, suggesting a role of RANBP2 in regulating innate immune cells [11]. In our two patients, genetic testing for RANBP2 mutations was negative. Unfortunately, other potential risk factors for ANE, except for genetic mutation, have not yet been clearly identified.

To date, the choice of strategy for best managing ANE remains controversial. Apart from supportive therapy aimed at treating seizures and intracranial hypertension, the “standard of care” may include antiviral agents (e.g., oseltamivir), immunoglobulins, and high-dose corticosteroids, either with methylprednisolone pulse therapy or intravenous dexamethasone. In particular, a correlation between the timely administration of steroid therapy (within 24 h of onset of symptoms) and a positive outcome has been reported in some studies [6]. Sadly, even though we rapidly started treatment with high-dose corticosteroids in our patients, their ultimate prognosis was poor.

In addition to early administration of corticosteroids and supportive care, the Infectious Diseases Society of America (ISDA) has proposed the use of high-dose oseltamivir for at least 14 days, as some recent studies reported a better outcome when the two drugs were combined [1,6]. However, greater consensus regarding the second line of treatment strategies is required. Based on the possible immune-mediated origin of ANE, some authors have suggested plasma exchange, immunosuppressants, and therapeutic hypothermia as anti-cytokine treatment [5,15,16]. In particular, anti-cytokine activity is one of the beneficial effects of therapeutic hypothermia in acute brain injury, and reduction in serum IL-6 and cytokine levels is associated with a better outcome [7].

Considering the critical involvement of cytokines in the pathogenesis of ANE, other therapeutic options include anti-interleukin-6 agents, such as tocilizumab, especially if administered in the acute phase of the disease (within 18–32 h of the onset of ANE) and in patients with high serum IL-6 levels [17]. Interestingly, IL-6 receptor blockade has been proven effective in the early stages of new-onset refractory status epilepticus and as an additional therapy in the treatment of refractory autoimmune encephalitis [17].

Finally, RANBP2 could be a promising therapeutic target in some genetic forms of ANE, suppressing the cytokine storm and the hyperinflammation associated with viral infection [11].

In our two patients, oseltamivir was not administered because viral triggering was not suspected upon admission, and the influenza virus was detected only after a few days. Despite high-dose corticosteroids and immunoglobulins being administered within the first days following the onset of ANE symptoms, the neurological outcome was poor in both cases.

At present, annual influenza vaccination in Italy is recommended for children from six months of age and is strongly recommended in patients with chronic diseases (e.g., severe asthma, cystic fibrosis), but vaccination is not mandatory in the pediatric and adult population [18]. The debate regarding whether influenza vaccination should be universally recommended for the pediatric population is still open, as can be seen in the two severe cases of ANE described above and the other influenza-associated forms of encephalopathy/encephalitis [19].

## 4. Conclusions

Acute necrotizing encephalopathy is a rare but severe complication of influenza virus infection, mainly affecting the pediatric population. Rapid recognition of neurological symptoms and suspicion of a viral trigger, particularly the influenza virus, may be crucial for the timely administration of effective therapy. Further research is needed to clarify the pathophysiology of ANE and establish the best therapeutic strategies to improve the outcome of such a catastrophic disease.

## Data Availability

The original data presented in the study are included in the article, further inquiries can be directed to the corresponding author.
